# Pyramidal cell activity levels affect the polarity of activity-induced gene transcription changes in interneurons

**DOI:** 10.1152/jn.00287.2018

**Published:** 2018-08-15

**Authors:** R. Ryley Parrish, Neela K. Codadu, Claudia Racca, Andrew J. Trevelyan

**Affiliations:** ^1^Institute of Neuroscience, Medical School, Newcastle University, Newcastle upon Tyne, United Kingdom; ^2^Columbia Translational Neuroscience Initiative, Department of Neurology, Columbia University, New York, New York

**Keywords:** epilepsy, epileptogenesis, homeostasis, MAPK pathway, parvalbumin

## Abstract

Changes in gene expression are an important mechanism by which activity levels are regulated in the nervous system. It is not known, however, how network activity influences gene expression in interneurons; since they themselves provide negative feedback in the form of synaptic inhibition, there exists a potential conflict between their cellular homeostatic tendencies and those of the network. We present a means of examining this issue, utilizing simple in vitro models showing different patterns of intense network activity. We found that the degree of concurrent pyramidal activation changed the polarity of the induced gene transcription. When pyramidal cells were quiescent, interneuronal activation led to an upregulation of glutamate decarboxylase 1 (*GAD1*) and parvalbumin (*Pvalb*) gene transcriptions, mediated by activation of the Ras/extracellular signal-related kinase mitogen-activated protein kinase (Ras/ERK MAPK) pathway. In contrast, coactivation of pyramidal cells led to an ionotropic glutamate receptor *N*-methyl-d-aspartate 2B-dependent decrease in transcription. Our results demonstrate a hitherto unrecognized complexity in how activity-dependent gene expression changes are manifest in cortical networks.

**NEW & NOTEWORTHY** We demonstrate a novel feedback mechanism in cortical networks, by which glutamatergic drive, mediated through the Ras/ERK MAPK pathway, regulates gene transcription in interneurons. Using a unique feature of certain in vitro epilepsy models, we show that without this glutamatergic feedback, intense activation of interneurons causes parvalbumin and glutamate decarboxylase 1 mRNA expression to increase. If, on the other hand, pyramidal cells are coactivated with interneurons, this leads to a downregulation of these genes.

## INTRODUCTION

The interplay between gene expression changes and neuronal activity is fundamental for normal brain function. Neuronal excitability is determined by which genes are expressed, and in turn, neuronal activity influences gene expression (Qiu and Ghosh 2008; West et al. 2002). These reciprocal interactions thus maintain functional stability and regulate metabolic requirements. There are other processes, though, that occur during development and learning, which rather than preserve the status quo, instead lead to long-lasting changes in brain networks (Beck and Yaari 2008; Karabanov et al. 2015). Similarly, various brain insults, including brain trauma, stroke, and infection, can lead to persistent pathological changes, such as the tendency to suffer epileptic seizures (Beck and Yaari 2008). This change is termed epileptogenesis and represents a major clinical problem because currently there is no prophylactic therapy (Pitkänen and Lukasiuk 2011). There remains, therefore, an urgent need to improve our understanding of how such changes are regulated in cortical networks.

Activity-dependent gene expression changes are often discussed in terms of neuronal activity homeostasis (Davis 2006, 2013; Marder and Prinz 2002). Homeostasis within the interneuronal population, however, may be more complex, because they themselves provide a different kind of negative feedback, in the form of synaptic inhibition. To illustrate this issue, consider the case where the entire network is active. If, in response, interneurons downregulate their excitability, this leads to a relative disinhibition of the network. If, on the other hand, homeostasis is manifested at the network level instead of at the cellular level, then a network-wide stimulation may result in a paradoxical response in the interneuronal population to increase their excitability. This latter case would represent a situation where the network’s needs are prioritized over the interneurons’ needs. These considerations would predict that activity-dependent changes in interneuronal gene expression are dictated by the level of concurrent pyramidal activity. To test this hypothesis, we utilized the fact that certain simple pharmacological manipulations of brain slice activity can induce very different patterns of epileptiform discharges. One manipulation (4-aminopyridine with glutamate receptor blockers; 4AP-GluX model) induces epileptiform activity only in the interneuronal population, in contrast to bathing slices in 0 Mg^2+^ artificial cerebrospinal fluid (ACSF), which triggers epileptiform activity in the entire neuronal population. We focused on the earliest detectable changes indicative of an activity-dependent gene expression response, which is in the mRNA transcript. We report here that the two patterns of cortical activation, which differ primarily in the level of pyramidal involvement, induced opposite changes in mRNA expression levels of glutamate decarboxylase 1 (*GAD1*) and parvalbumin (*Pvalb*) genes. These opposing gene expression changes appear to be downstream of the glutamatergic-activated Ras/extracellular signal-related kinase mitogen-activated protein kinase (Ras/ERK MAPK) pathway. Collectively, these data suggest that activity-dependent changes in gene expression are influenced by interactions between different cell classes.

## METHODS

### 

#### Tissue preparation.

Procedures were performed according to the guidelines of the Home Office UK Animals (Scientific Procedures) Act of 1986. Adult male (2–4 mo) wild-type (C57/BL6) or PV-Cre Het mice were used in this study. Mice were decapitated, and brains were removed and stored in cold cutting solution (in mM): 3 MgCl_2_, 126 NaCl, 26 NaHCO_3_, 3.5 KCl, 1.26 NaH_2_PO_4_, and 10 glucose. For local field potential (LFP) recordings, 400-μm horizontal sections containing the neocortex, entorhinal cortex, and hippocampus were made, using a Leica VT1200 vibratome (Nussloch, Germany). Slices were then transferred to an interface holding chamber and incubated for 1–2 h at room temperature in ACSF containing (in mM): 2 CaCl_2_, 1 MgCl_2_, 126 NaCl, 26 NaHCO_3_, 3.5 KCl, 1.26 NaH_2_PO_4_, and 10 glucose. For patch-clamp experiments, 350-μm coronal sections were made but were stored in a submerged holding chamber for 1–4 h before experimentation. All of the solutions were bubbled continuously to saturate with carboxygen (95% O_2_ and 5% CO_2_).

Extracellular field recordings were performed using interface recording chambers. Horizontal brain slices [approximately equivalent to *plate 155* in Franklin and Paxinos (2008)] were placed in the recording chamber perfused with ACSF or modified to induce epileptiform activity [0 Mg^2+^/4AP (100 μM, Sigma-Aldrich)/gabazine (10 mM, Tocris)] or to include various pharmacological agents [50 µM d-2-amino-5-phosphonovalerate (AP5, Hello-Bio, Bristol, UK), 20 µM NBQX disodium salt (Hello Bio), 1 μM Ro-25-6981 (Santa Cruz Biotechnology, Heidelberg, Germany), and 20 µM U0126 (New England Biolabs, Hitchin, UK)]. Since U0126 and Ro-25-6981 were both prepared in 0.1% dimethyl sulfoxide (DMSO), the parallel control experiments were also run using 0.1% DMSO (but omitting U0126 and Ro-25-6981).

Recordings were obtained using normal ACSF-filled 1–3 MΩ borosilicate glass microelectrodes (GC120TF-10; Harvard Apparatus, Cambridge, UK) placed in deep layers of neocortex [corresponding to temporal association area, *plate 155* (Franklin and Paxinos 2008). Experiments were performed at 33–36°C. The solutions were perfused at the rate of 3 ml/min. Waveform signals were acquired using BMA-931 biopotential amplifier (Dataq Instruments, Akron, OH), Micro 1401-3 ADC board (Cambridge Electronic Design, Cambridge, UK), and Spike2 software (v. 7.10, Cambridge Electronic Design). Signals were sampled at 10 kHz, amplified (gain: 500), and bandpass filtered (1–3,000 Hz). A CED4001-16 Mains Pulser (Cambridge Electronic Design) was connected to the events input of CED micro 1401-3 ADC board and used to remove 50-Hz hum offline. Extracellular field recordings were analyzed to detect events using a custom-written code in Matlab2015b (MathWorks, Natick, MA).

#### Viral injections.

PV-Cre pups were injected with AAV5.Syn.Flex.tdTomato, purchased from the University of Pennsylvania vector core. Injections were performed on the day of birth. Pups were set on a stereotaxic frame and anesthetized with isoflurane, following application of EMLA cream (2.5% Lidocaine and 2.5% Prilocaine) to the left top of their heads. Injections were made using 10 μl of Hamilton syringes with a beveled 36-gauge needle (World Precision Instruments), unilaterally into the lateral ventricle and overlying cortical plate, at ~1 mm anterior to lambda and 1 mm lateral to the midline into the left hemisphere, starting at 1.7 mm deep from the top of the dura mater, for a total of four separate 50-nl injections, the deepest first and coming up 0.3 mm for each subsequent injection. Approximately 200 nl (∼1,000 viral particles) was injected into the left hemisphere over a 10-min period. Pups were monitored until they awoke following the procedure and then returned to their home cage. These neonatal injections produced widespread cortical expression of tdTomato into the parvalbumin positive (PV) cells.

#### Patch-clamp recordings.

Slices were perfused at 3–5 ml/min and heated to 33–34°C. Whole cell data were acquired using pClamp software, Multiclamp 700B, and Digidata acquisition board (Molecular Devices, Sunnyvale, CA). Whole cell and cell-attached recordings were made using 4- to 7-MΩ pipettes. Pipettes were filled with a KMeSO_4_-based internal solution containing (in mM) 125 KMeSO_4_, 6 NaCl, 10 HEPES, 2.5 Mg-ATP, 0.3 Na_2_-GTP, and 5% (wt/vol) biocytin. For the voltage clamp recordings, 5 mM QX-314 [*N*-(2,6-dimethylphenylcarbamoylmethyl) triethylammonium bromide; Sigma-Aldrich] was added to the internal solution. Osmolarity and pH of all the internal solutions used were adjusted to 284 mOsm and 7.4, respectively. PV cells were identified using tdTomato for targeted cell-attached recordings.

#### Tissue collection.

After 1 h of recordings, tissue was immersed in Ambion RNAlater solution (Thermo Fisher Scientific, Cramlington, UK), the neocortex was subdissected away from the entorhinal cortex and the hippocampus and then stored in RNAlater solution, at 4°C until the RNA was extracted (see approximate area indicated in the schematic in Fig. 3*Aiii*).

#### RNA preparations.

RNA was extracted using the RNAqueous-Micro Total RNA Isolation Kit (Thermo Fisher Scientific) and eluted in 15 µl of provided elution buffer. Residual genomic DNA was removed using reagents supplied in the above-mentioned kit and done according to the manufacturer’s instructions. Concentration and purity of RNA were determined using a BioDrop DUO (BioDrop, Cambridge, UK). RNA was then stored at −80°C until the cDNA library preparation.

#### Real-time PCR.

Samples were normalized to 100 ng for cDNA library preparation. cDNA synthesis was performed using the Applied Biosystems High-Capacity cDNA Reverse Transcription Kit (Thermo Fisher Scientific) in a Thermo Hybaid PCR Express Thermal Cycler (Thermo Hybaid, Ashford, UK) at a total volume of 20 µl. The cDNA was diluted 1:4 for the neocortical samples with nuclease-free H_2_O. The following primers were used for the real-time (RT)-PCR: *Pvalb* (For: attgaggaggatgagctggg, Rev: cgagaaggactgagatgggg), *GAD1* (For: gcctggaagagaagagtcgt, Rev: tccccgttcttagctggaag), glyceraldehyde-3-phosphate dehydrogenase (*Gapdh*) (For: acctttgatgctggggctggc, Rev: gggctgagttgggatggggact). q-PCR amplifications were performed on an Applied Biosystems Step One Plus RT PCR system (Thermo Fisher Scientific) at 95°C for 10 min, and then 40 repeats of the following cycle: 95°C for 10 s, followed by 58°C for 30 s and 95°C for 15 s. This protocol was immediately followed by the melt curve starting at 95°C for 15 s, 60°C for 1 min, 95°C for 15 s, and, finally, held at 4°C. All samples were run in duplicate at a primer molarity of 10 μmol/l. *Pvalb* and *GAD1* were compared with *Gapdh*. Expression of *Gapdh* was unchanged across all treatment groups (data not shown). Cycle threshold (C_T_) values were analyzed using the comparative C_T_ method to calculate differences in gene expression between samples. Melt curve analysis was used to confirm there was a single product from each primer, and samples were run on a DNA agarose gel to confirm expected product size and melt curve results.

#### Statistical analysis.

Relative mRNA fold changes for all genes were analyzed using the comparative C_T_ method. Due to a lack of homogeneity of variance between groups (Brown-Forsythe test and Bartlett's test), mRNA expression data were first transformed using a square root transformation. Square root transformation of mRNA data resulted in a nonsignificant Brown-Forsythe test and Bartlett’s test. Transformed mRNA data and electrophysiology data of three or more groups were anaylzed using a one-way analysis of variance (ANOVA) with Tukey’s post hoc test. All mRNA data are displayed as they were analyzed, as the square root of the relative value. Electrophysiology data of only two groups were analyzed using Student’s *t*-test. Significance was set at *P* ≤ 0.05 for all analyses. Figures of mRNA expression and event detection, along with all statistics, were done using GraphPad Prism (GraphPad Software, La Jolla, CA). Figures of electrophysiology traces were created in Igor Pro (WaveMetrics, Lake Oswego, OR).

## RESULTS

### 

#### Different epileptiform activity induced by 0 Mg^2+^ and 4-aminopyridine.

**Fig. 1.**
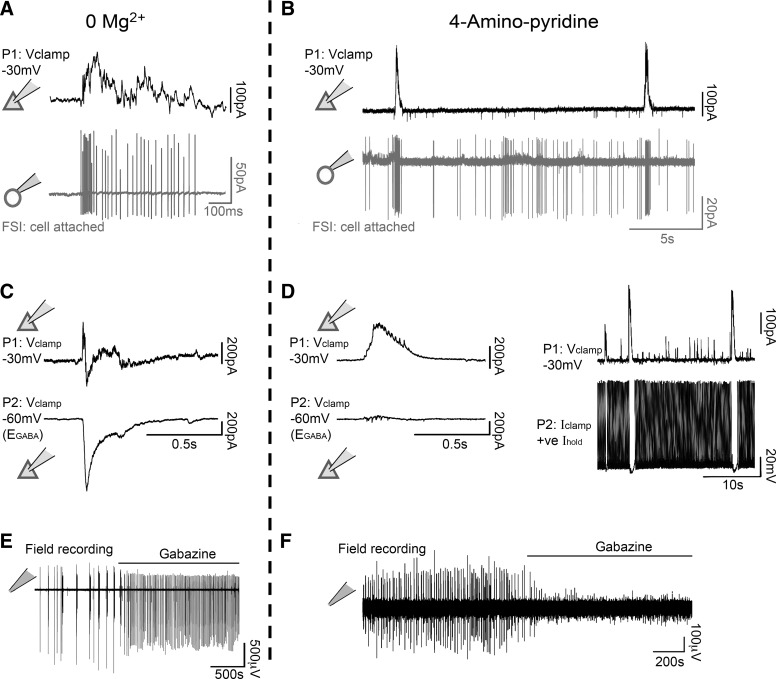
Differential neuronal involvement in epileptiform discharges induced by 0 Mg^2+^ or by 4-aminopyridine (4AP). *A*: paired recordings of neighboring layer 5 pyramidal cells (P; whole cell, voltage clamp at −30 mV) and fast-spiking interneurons (FSI; cell attached) recorded during epileptiform bursts induced by bathing brain slices in 0 Mg^2+^ (representative example; *n* = 6 slices). *B*: similar paired recordings shortly after bath application of 100 µM 4AP, also showing very intense bursting of fast-spiking interneurons coincident with the timings of γ-aminobutyric acid (GABA)ergic barrages onto the pyramidal cells (representative example; *n* = 4 slices). *C*: paired voltage clamp whole cell recordings (P1 held at −30 mV, and P2 at −60 mV, close to E_GABA_) from closely apposed (<50 µm) layer 5 pyramidal cells during transient interictal discharges in 0 Mg^2+^ artificial cerebrospinal fluid (ACSF) (representative example; *n* = 3 slices). Note particularly the large downward deflection in P2, demonstrating the presence of a large glutamatergic component in these events. *D*: similar recordings in 4AP (representative example; *n* = 3 slices). Note the almost complete absence of any glutamatergic activity in the P2 recording, consistent with the key feature of the *right* panels, which is that, in current clamp, with a depolarizing drive to induce firing, the GABAergic barrages can veto even this artificial excitation.: extracellular recordings of local field potentials (LFPs) in layer 5 in 0 Mg^2+^ (representative example; *n* = 3 slices; *E*) and 4AP with glutamate receptor blockers (4AP-GluX) models (representative example; *n* = 3 slices; *F*). Note the opposite effects of gabazine, which leads to an increase in electrographic activity in the 0 Mg^2+^ model (*E*) but a blockade of it in the 4AP-GluX model (*F*).

**Fig. 2.**
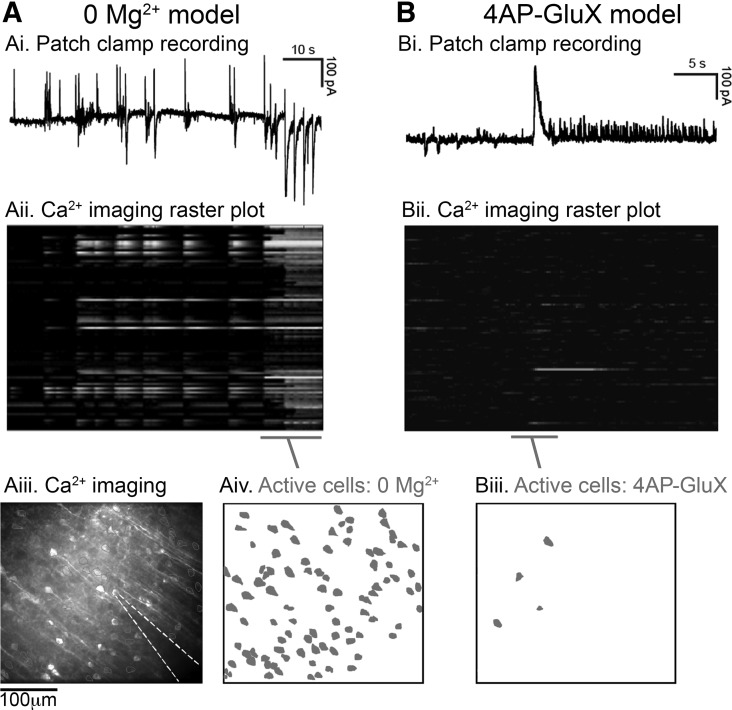
Simple in vitro pharmacological manipulations can be used to induce disparate patterns of epileptiform discharges involving varying decreases of cellular activity. *A*: activity patterns in the 0 Mg^2+^ model, as manifested in whole cell recordings of layer 5 pyramidal cells in voltage clamp mode (−30 mV holding potential) with simultaneous Ca^2+^ network imaging (*Aii–iv*). Note the widespread activation (95 active cells shown in red, *Aiv*). *B*: continued recording of the same cell and field of view after switching to the 4-aminopyridine with glutamate receptor blockers (4AP-GluX) model. Note the minimal cellular recruitment (4 active cell) during what appear to be large electrographic events as recorded in patch clamp mode.

Epileptiform activity can be induced in brain slices by a variety of simple pharmacological manipulations, including removal of Mg^2+^ ions from the bathing medium or addition of the K^+^ channel blocker 4-aminopyridine (4AP) (Swartzwelder et al. 1987; Ives and Jefferys 1990). Electrophysiological recordings in both cases showed evidence of intense and widespread neuronal activation (Fig. 1). Ca^2+^ network imaging and patch clamp studies, on the other hand, presented a more detailed and nuanced view of local neuronal involvement. Importantly, both models showed very intense and repeated activation of the interneuronal population (Fig. 1, *A* and *B*) including fast-spiking interneurons. As such, these paradigms present a means to examine how this critical class of interneuron may react to induced intense activation. The two models differ markedly, however, in the level of pyramidal activation. In the 0 Mg^2+^ model, the earliest discharges have a predominantly interneuronal involvement but with limited pyramidal cell activity; however, this changes with the occurrence of the start of full ictal events, and thereafter, all neurons are activated in most, if not every discharge (Fig. 1*C*) (Trevelyan et al. 2006; Parrish et al. 2018). In contrast, the early activity in the 4AP model appears to lack any significant pyramidal involvement; voltage clamp recordings show minimal deflections (Fig. 1*D*), when the cell is held at close to the GABAergic reversal potential (contrast this with equivalent recordings in 0 Mg^2+^, Fig. 1*C*), whereas, if the pyramidal cell is held in current clamp, there is a pronounced inhibition of firing during the discharges (Fig. 1*D*, *right*). Multiple studies have noted that the 4AP model can subsequently progress to full ictal activation (including pyramidal involvement) (Galvan et al. 1982; Avoli et al. 2002). This early activity pattern can be stabilized, however, by adding the glutamatergic blockers NBQX and AP5, to the 4AP bathing medium (4AP-GluX) (Avoli et al. 2002; Bohannon and Hablitz 2018). If one then applies the γ-aminobutyric acid A (GABA_A_) receptor antagonist gabazine in the 4AP-GluX, there is a rapid cessation of all electrographic activity recorded extracellularly (Fig. 1*F*), in stark contrast to what happens in the 0 Mg^2+^, where gabazine induces a pronounced escalation of activity (Fig. 1*E*). These two different patterns of activity can be demonstrated from a single slice (Fig. 2 and Supplemental Movies S1, S2, and S3; supplemental materials for this article are available on the *Journal of Neurobiology* website), where washout of Mg^2+^ ions leads to widespread neuronal activation, with 95 active cells during a seizure event (Fig. 2*A* and Supplemental Movie S1). In contrast, following Mg^2+^ ions being washed back in, and with the addition also of 4AP-GluX, we recorded only induced pluripotent stem cells (iPSCs) in the postsynaptic pyramidal cell (Fig. 2*B*), and, with minimal network activity, just four active cells at the times of the synaptic bursts (Fig. 2*B* and Supplemental Movies S2 and S3).

#### Pyramidal coactivation alters gene transcription changes in PV interneurons.

**Fig. 3.**
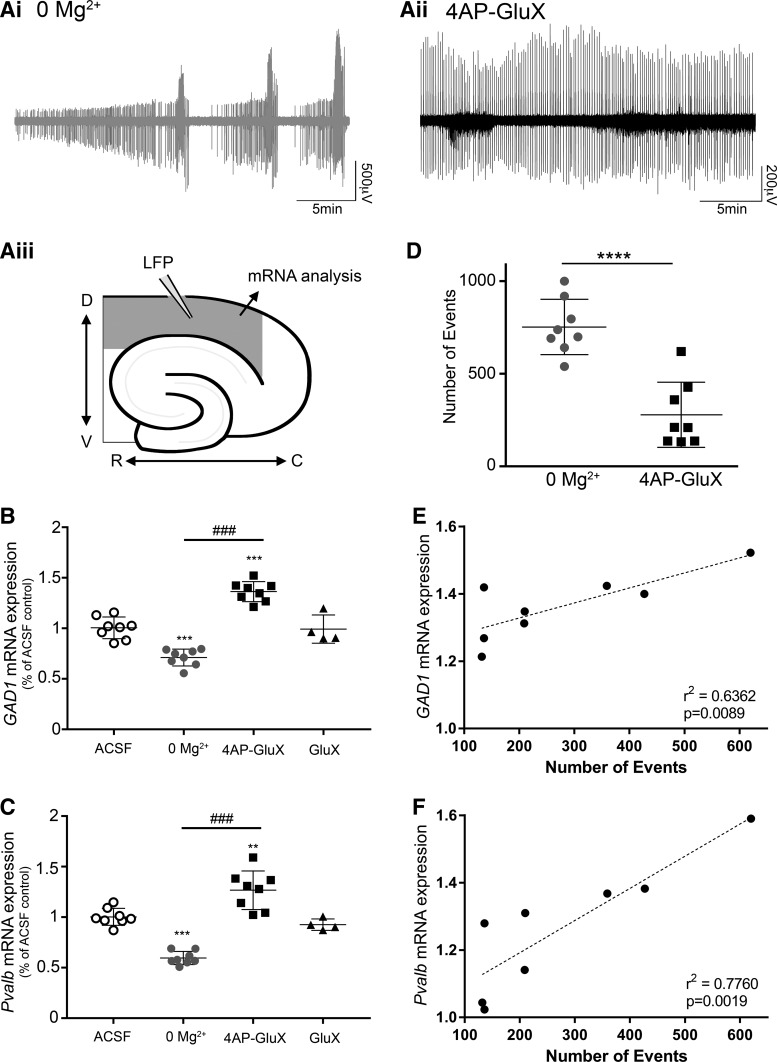
Glutamate decarboxylase 1 (*GAD1*) and parvalbumin (*Pvalb*) gene transcriptions are regulated differently when interneurons are activated in isolation vs. when activated with the whole network in tandem.***Ai*: r**epresentative epileptiform discharges from the 0 Mg^2+^ model. *Aii*: representative epileptiform discharges from the 4-aminopyridine with glutamate receptor blockers (4AP-GluX) model. *Aiii*: schematic to show the location of the electrode recording the local field potential (LFP) and the source of the tissue for analysis of the neocortical mRNA transcription analyses. *B*: *GAD1* mRNA expression in the neocortex was significantly downregulated due to total network activation (0 Mg^2+^). Oppositely, *GAD1* mRNA expression was significantly increased in the neocortex due to selective interneuronal activation [4AP-GluX, *n* = 8 for artificial cerebrospinal fluid (ACSF), 0 Mg^2+^, and 4AP-glutamate receptor (GluR) blocker groups; *n* = 4 for GluR blockers group; ***P < 0.001 relative to ACSF; ^###^*P* < 0.001 between relevant experiment groups). *C*: *Pvalb* mRNA expression was significantly decreased due to network activation (0 Mg^2+^). Oppositely, its expression was significantly increased due to interneurnal activity (4AP-GluX, *n* = 8 for ACSF, 0 Mg^2+^, and 4AP-GluR blockers groups; *n* = 4 for GluR blocker group; **P < 0.01 relative to ACSF, ***P < 0.001 relative to ACSF; ^###^*P* < 0.001 between relevant experiment groups). *D*: quantification of the number of events produced between induction of total network activity using the 0 Mg^2+^ model and the events produced using the 4AP-GluX model in the neocortex (*n* = 8; ****P < 0.0001, *P < 0.05). *E*: *GAD1* mRNA expression in the neocortex is activity dependent following the selective interneuronal activation (4AP-GluX; *n* = 8, *r*^2^ = 0.6362, *P* = 0.0089). *F*: *Pvalb* mRNA expression in the neocortex is activity dependent following the selective interneuronal activation (4AP-GluX; *n* = 8, *r*^2^ = 0.7760, *P* = 0.0019).

Aside from what these models might tell us about epilepsy, they have an added utility, because, although both show intense interneuronal activation, the level of pyramidal involvement is very different: high in 0 Mg^2+^ and low in 4AP. Thus, a comparison of these models allowed a direct test of our hypothesis, that gene expression in the interneuron network is affected by the level of pyramidal cell activity. We examined two target genes, *GAD1* and *Pvalb*, which are selectively expressed in inhibitory interneurons, and which are also known to be dynamically regulated due to differential stimuli (Donato et al. 2013; Hanno-Iijima et al. 2015). It is also relevant that PV-expressing interneurons are an important network component in both the 0 Mg^2+^ model and the 4AP model (Fig. 1) (Cammarota et al. 2013; Sessolo et al. 2015). We exposed brain slices to one of four different bathing media [1: control ACSF; 2: 0 Mg^2+^ ACSF; 3: 4AP-GluX; 4: glutamatergic blockers in control ACSF (GluX)] for 1 h while recording activity using extracellular electrodes (Fig. 3*A*). In brain slices with activation oly of interneurons (4AP-GluX), we found highly significant increases in mRNA expression of both the *GAD1* and *Pvalb* genes (Fig. 3, *B* and *C*). In contrast, when interneurons were coactive with pyramidal cells, (0 Mg^2+^ ACSF), both genes showed the opposite change, with a highly significant decrease (Fig. 3, *B* and *C*). Furthermore, there was a highly significant difference between the levels of mRNA expression of these genes between the two models. Bathing slices in normal ACSF with glutamatergic blockers resulted in no change in *GAD1* or *Pvalb* mRNA expression (Fig. 3, *B* and *C*).

Different brain slices showed variation in the degree of epileptiform activation, with the 0 Mg^2+^ model typically inducing more discharges than the 4AP model (Fig. 3*B*). This factor, though, cannot explain the differences in gene expression between the groups, first, because the expression patterns were in opposing directions; second, in the 4AP-GluX model there was a highly significant correlation with the degree change in gene expression and the amount of activity (Fig. 3, *E* and *F*), suggesting that, if these slices had shown similar levels of activation to the 0 Mg^2+^ model, the difference between the models might have been even greater. The 0 Mg^2+^ model showed no correlation between the number of local field potential (LFP) events detected in the neocortex with the decrease in expression of both *GAD1* and *Pvalb* mRNA (*GAD1*, *r*^2^ = 0.004, *P* = 0.4424; *Pvalb*, *r*^2^ = 0.117, *P* = 0.2036).

#### GAD1 and Pvalb transcription is modulated by GluN2B-containing NMDA receptors and Ras/ERK MAPK pathway.

Collectively, these data suggest that the gene-transcription response in interneurons is critically affected by whether or not there is coactivation of pyramidal cells. We hypothesized that glutamatergic drive onto the interneurons, in the 0 Mg^2+^ model, might influence the nature of the gene transcription profile. One candidate for mediating this effect is the Ras/ERK MAPK pathway, since it is known to be activity dependent (Thomas and Huganir 2004). Furthermore, its regulation is directly tied to fluctuations of Ca^2+^ concentrations and is also surprisingly sensitive to the mechanism of Ca^2+^ entry into the cell (Thomas and Huganir 2004). Specifically, Ca^2+^ entering through GluN2B-containing NMDA receptors (NMDARs), which are primarily extrasynaptic, deactivates the Ras/ERK MAPK pathway, whereas other modes of Ca^2+^ entry into the cell, such as through GluN2A-containing NMDARs and voltage-gated Ca^2+^ channels result in activation of the Ras/ERK MAPK pathway. *GAD1* and *Pvalb* expression can also be regulated by Ca^2+^ fluctuations either through NMDARs and/or voltage-gated calcium channels (Cohen et al. 2016; Hardingham and Bading 2010; Kim et al. 2005), and their expression has been demonstrated in some cases to be downstream of the Ras/ERK MAPK pathway (Hanno-Iijima et al. 2015; Patz et al. 2003, 2004). We therefore investigated how Ca^2+^ entry might explain the divergent patterns of gene transcription changes seen in the two models.

**Fig. 4.**
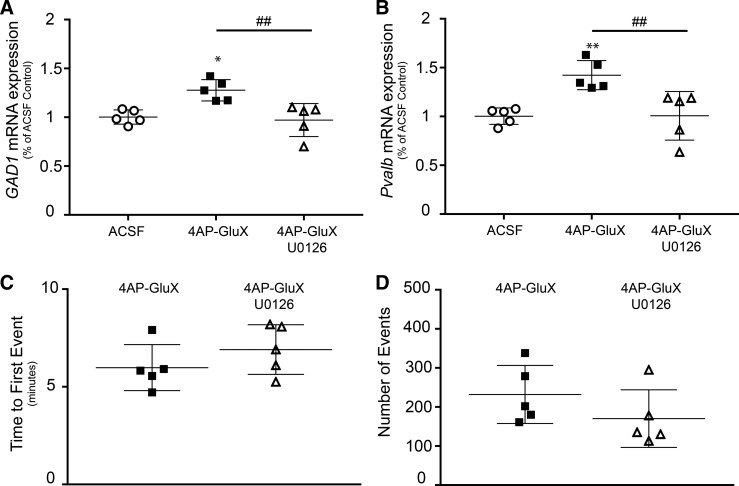
Upregulation of glutamate decarboxylase 1 (*GAD1*) and parvalbumin (*Pvalb*) gene transcription is blocked by MAPK *kinase* (MEK) inhibition in the 4-aminopyridine with glutamate receptor blockers (4AP-GluX) model. *A*: increase of *GAD1* mRNA expression due to selective interneuronal activation (4AP-GluX) was blocked by application of the MEK inhibitor U0126 (*n* = 5; *P < 0.05 relative to ACSF; ^##^*P* < 0.01 between relevant experiment groups). *B*: increase of *Pvalb* mRNA expression due to selective interneuronal activation (4AP-GluX) was blocked by application of MEK inhibitor (*n* = 5; **P < 0.01 relative to ACSF; ^##^*P* < 0.01 between relevant experiment groups). *C*: MEK inhibition did not significantly alter time to first event (*n* = 5). *D*: MEK inhibition did not significantly alter the activity generated in the 4AP-GluX model (*n* = 5).

The increased gene transcription seen in the 4AP-GluX model was prevented by U0126 (Fig. 4), which suppresses the Ras/ERK MAPK pathway through its action on MEK1. Importantly, this occurs without altering the intensity of epileptiform activity, quantified by time to first event (Fig. 4*C*) and total number of events over the hour-long recording (Fig. 4*D*).

**Fig. 5.**
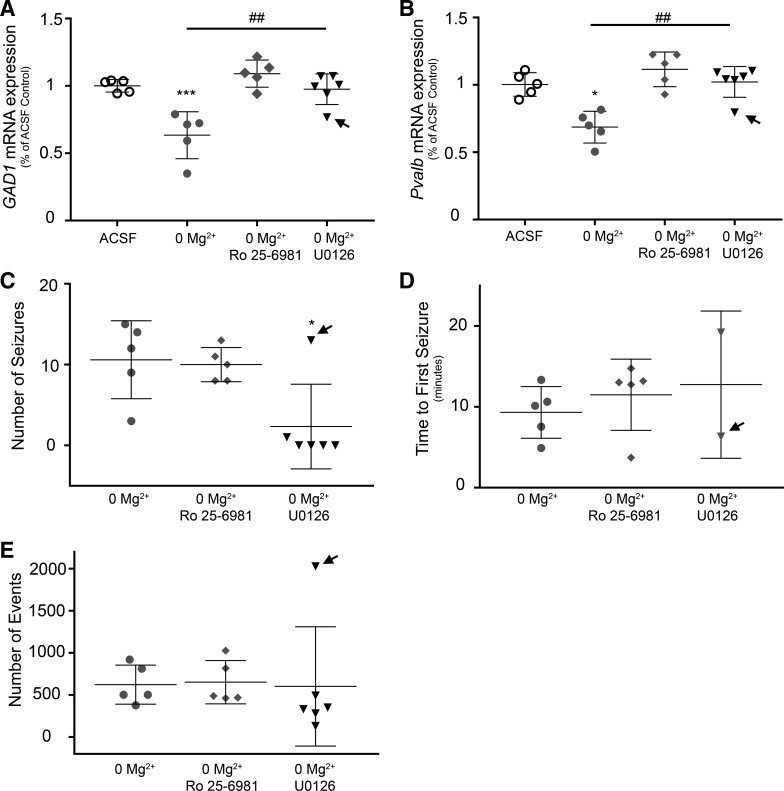
Decrease in glutamate decarboxylase 1 (*GAD1*) and parvalbumin (*Pvalb*) gene transcription is reversed by blocking extrasynaptic GluN2B-containing *N*-methyl-d-aspartate (NMDA) receptors in the 0 Mg^2+^ model. *A*: downregulation of neocortical *GAD1* mRNA expression due to total network activity (0 Mg^2+^) was blocked by specific extrasynaptic GluN2B-containing NMDA receptor inhibitor Ro25-6981 (*n* = 5–6; ***P < 0.001 relative to ACSF; ^##^*P* < 0.01 between relevant experiment groups). *B*: decrease of *Pvalb* mRNA expression due to total network activity was blocked by extrasynaptic GluN2B-containing NMDA receptor inhibitor in the neocortex (*n* = 5–6; *P < 0.05 relative to ACSF; ^##^*P* < 0.01 between relevant experiment groups). *C*: blocking extrasynaptic GluN2B-containing NMDA receptors had no effect on number of seizures. Inhibition of U0126, a nonselective MAPK *kinase* (MEK) inhibitor significantly reduced seizures (*n* = 5–6; *P < 0.05 relative to 0 Mg^2+^ and 0 Mg^2+^ + Ro25-6981). *D*: blocking the extrasynaptic GluN2B containing NMDA receptors had no effect on ictal start time (*n* = 5). *E*: blocking of extrasynaptic GluN2B-containing NMDA receptors (Ro25-6981) and blocking the MEK pathway had no significant effect on total activity generated by removal of Mg^2+^ ions (*n* = 5–6).

U0126 also prevented the change in gene transcription in the 0 Mg^2+^ model (Fig. 5, *A* and *B*). The interpretation of this result, though, is complicated by the fact that U0126 significantly reduced the level of neuronal activity (Fig. 5, *C*–*E*), preventing virtually all seizure-like events in five of six slices, consistent with the findings from a previous study (Merlo et al. 2004). Interestingly, there continued to be a high level of interictal activity (Fig. 5*E*). This activity, similarly to the 4AP-induced discharges, was dominated by interneuronal firing and low pyramidal involvement (Schevon et al. 2012). Notably, in the one slice in U0126 that did show multiple seizure-like events (that is, high pyramidal involvement; Fig. 5, *C*–*E*, arrows), the mRNA expression levels of *GAD1* and *Pvalb* were comparable with the data from the 0 Mg^2+^ model without U0126, consistent with the interpretation that the key trigger for reducing the *Pvalb* and *GAD1* mRNA were the episodes of intense pyramidal activation.

Blocking GluN2B-containing NMDARs with Ro25-6981 prevented the decrease in *GAD1* and *Pvalb* expression seen in the 0 Mg^2+^ model (Fig. 4, *A* and *B*), but in contrast to the U0126 result, Ro25-6981 did so without altering the intensity of epileptiform activity, quantified by the total number of seizures (Fig. 4*C*), time to first seizure event (Fig. 4*D*), and total number of events (Fig. 4*E*). Thus, its antagonistic effect was downstream of the increase in neuronal activity. These data suggest that decreased neocortical *GAD1* and *Pvalb* mRNA expression was induced by Ca^2+^ entry via the GluN2B-containing NMDARs, presumably, due to glutamate spillover and activation of the extrasynaptic NMDARs. Taken together, these data suggest a novel feedback mechanism regulating interneuronal transcript expression, based on the glutamatergic drive they receive.

## DISCUSSION

**Fig. 6.**
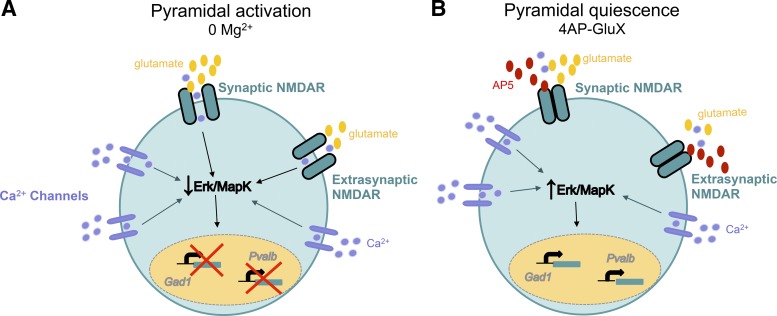
Model of mechanisms driving the differential transcription of glutamate decarboxylase 1 (*GAD1*) and parvalbumin (*Pvalb*) genes in parvalbumin protein (PV) cells in the 2 different types of epileptiform activity. *A*: full network activation (pyramidal activation; 0 Mg^2+^) leads to spillover of glutamate, activating extrasynaptic GluN2B-containing *N*-methyl-d-aspartate receptors (NMDAR), resulting in downregulation of *GAD1* and *Pvalb* mRNA expression. *B*: following 4-aminopyridine (4AP) with glutamate receptor (4AP-GluX) blockers (PV cell activation only, pyramidal quiescence), Ca^2+^ transients are still produced within the PV cells, resulting in physiological levels of Ca^2+^ and activation of the extracellular signal-related kinase mitogen-activated protein kinase (ERK MAPK) pathway, which is upstream of *GAD1* and *Pvalb* mRNA expression.

We have described two experimental conditions sharing a common feature of sustained intense bursting of interneurons but differing in the degree of pyramidal involvement and which lead to divergent gene transcription changes. The difference between the two conditions appears to be whether or not the pyramidal cell population is coactive. We have further shown how these gene expression responses are mediated via Ca^2+^ entry through GluN2B-containing NMDARs and downstream of the Ras/ERK MAPK pathway. Figure 6 illustrates the putative chain of network and molecular events underlying the two different gene expression outcomes. The Ras/ERK MAPK pathway is regulated by activity-dependent Ca^2+^ entry (Thomas and Huganir 2004), which in turn influences *GAD1* and *Pvalb* mRNA expression and determines the direction of their expression. Ca^2+^ influx has a bidirectional effect on the activation of the Ras/ERK MAPK pathway, depending on whether entry occurs via GluN2A-containing NMDARs (mainly synaptic) or GluN2B-containing NMDARs (mainly extrasynaptic): GluN2A entry activates the Ras/ERK MAPK pathway, whereas GluN2B entry downregulates it (Hardingham and Bading 2010; Kim et al. 2005). Seizure-like levels of neuronal activation, particularly, are likely to favor entry through the extrasynaptic GluN2B-containing NMDARs (Hardingham and Bading 2010), which explains why the 0 Mg^2+^ paradigm results in reduced *GAD1* and *Pvalb* mRNA expression. This interpretation is consistent with other studies showing that blocking the Ras/ERK MAPK pathway decreases *GAD1* expression (Patz et al. 2004) and, second, that *GAD1, GAD2*, and *Pvalb* expression are activity dependent and downstream of Ca^2+^ entry (Cohen et al. 2016), and activation of the Ras/ERK MAPK pathway (Hanno-Iijima et al. 2015; Patz et al. 2003). An interesting parallel with our data is that the direction of change of PV and GAD67 (the protein product of *GAD1*) can also be dictated by different behavioral experiences (Donato et al. 2013), although this study did not relate these changes to the involvement of pyramidal cells or the molecular pathways that transduce this interaction.

Blockade of Ras/ERK MAPK has previously been shown to reduce 4AP seizure-like events (Merlo et al. 2004), and we confirm this in the 0 Mg^2+^ model, although, notably in both models, the repeated discharges associated with interneuronal bursts (interictal events) are not suppressed. This suggests that interneurons and pyramidal cells show different Ras/ERK MAPK pathway behavior, which is further supported by reports that seizure activity induces activation of this pathway in pyramidal cells (Merlo et al. 2004) but inhibits it in GAD-positive interneurons (Choi et al. 2007).

Our findings represent an important nuance to the conventional paradigm of neuronal activity homeostasis, suggesting the possibility that, when there is intense interneuronal activation alone, they show one pattern of gene transcription change; but when there is widespread network activation, then their response is modulated by the concurrent pyramidal activity. Previous paradigms examining changes in neuronal firing rate typically have done so over periods of days (Turrigiano et al. 1994), and we did not see any evidence of a reactive drop in activity within the 1-h recordings. The level of *GAD1* mRNA expression, however, is likely to influence the pattern of feedback inhibition within the network. Our findings are thus very relevant to epileptogenesis, the protracted process whereby cortical networks undergo extensive reorganization leading to the occurrence of repeated spontaneous seizures. Although many researchers have documented a wide range of changes associated with this process (Dengler et al. 2017; Pitkänen and Engel 2014; Terrone et al. 2016), we still do not know which are the pivotal cellular changes (Pitkänen et al. 2015), and consequently, we still lack the scientific grounding to develop rational antiepileptogenic therapies. An important clinical observation is that various triggers of epileptogenesis, such as cerebrovascular accidents or trauma, lead to epilepsy only in a proportion of cases. This inherent binary variability is also seen in our gene transcription studies because our results imply that a linear increase in pyramidal involvement in pathological network events will, at some threshold, produce a switch from upregulation to downregulation of mRNA expression of certain genes. Our experiments looked at just two key genes, *GAD1 and Pvalb*, but epileptogenesis has been associated with changes in many thousands more (Hansen et al. 2014; Lachos et al. 2011; Rakhade et al. 2007), arising from altered epigenetic mechanisms such as DNA methylation (Miller-Delaney et al. 2012; Ryley Parrish et al. 2013). However, this proof-of-principle study serves to emphasize the importance of investigating cell class-specific gene changes, particularly with respect to variable interneuronal responses, during epileptogenesis (e.g., Hortopan et al. 2010; Jiang et al. 2016; Schwaller et al. 2004).

## GRANTS

This work was supported by Epilepsy Research UK and the Medical Research Council (UK), and a Schaefer Scholarship Award to A. J. Trevelyan.

## DISCLOSURES

No conflicts of interest, financial or otherwise, are declared by the authors.

## AUTHOR CONTRIBUTIONS

R.R.P. and A.J.T. conceived and designed research; R.R.P., N.K.C., and A.J.T. performed experiments; R.R.P. and A.J.T. analyzed data; R.R.P., C.R., and A.J.T. interpreted results of experiments; R.R.P., C.R., and A.J.T. prepared figures; R.R.P. and A.J.T. drafted manuscript; R.R.P., N.J.C., C.R., and A.J.T. edited and revised manuscript; R.R.P., N.K.C., C.R., and A.J.T. approved final version of manuscript.
